# Knowledge, attitudes, and preferences of healthy young adults regarding advance care planning: a focus group study of university students in Pittsburgh, USA

**DOI:** 10.1186/s12889-015-1575-y

**Published:** 2015-02-27

**Authors:** Dio Kavalieratos, Natalie C Ernecoff, Jessica Keim-Malpass, Howard B Degenholtz

**Affiliations:** Section of Palliative Care and Medical Ethics, Division of General Internal Medicine, University of Pittsburgh School of Medicine, 230 McKee Place, Suite 600, Pittsburgh, PA 15213 USA; Department of Behavioral and Community Health Sciences, Graduate School of Public Health, University of Pittsburgh, Pittsburgh, PA USA; School of Nursing, University of Virginia, Charlottesville, VA USA; Department of Health Policy and Management, Graduate School of Public Health, University of Pittsburgh, Pittsburgh, PA USA

**Keywords:** Advance care planning, Young adults, Qualitative, Health services research

## Abstract

**Background:**

To date, research and promotion regarding advance care planning (ACP) has targeted those with serious illness or the elderly, thereby ignoring healthy young adults. The purpose of this study was to explore young adults’ knowledge, attitudes, and preferences regarding advance care planning (ACP) and medical decision-making. Further, we aimed to understand the potential role of public health to encourage population-based promotion of ACP.

**Methods:**

Between February 2007 and April 2007, we conducted six focus groups comprising 56 young adults ages 18–30. Topics explored included (1) baseline knowledge regarding ACP, (2) preferences for ACP, (3) characteristics of preferred surrogates, and (4) barriers and facilitators to completing ACP specific to age and individuation. We used a qualitative thematic approach to analyze transcripts.

**Results:**

All participants desired more information regarding ACP. In addition, participants expressed (1) heterogeneous attitudes regarding triggers to perform ACP, (2) the opinion that ACP is a marker of individuation, (3) the belief that prior exposure to illness plays a role in prompting ACP, and (4) an appreciation that ACP is flexible to changes in preferences and circumstances throughout the life-course.

**Conclusion:**

Young adults perceive ACP as a worthwhile health behavior and view a lack of information as a major barrier to discussion and adoption. Our data emphasize the need for strategies to increase ACP knowledge, while encouraging population-level, patient-centered, healthcare decision-making.

## Background

Advance care planning (ACP) is a process that allows competent individuals to plan for future medical care [1,2]. ACP generally takes one of two forms: 1) the advance directive, or “living will”, a mechanism that allows individuals to catalog their preferences for future healthcare; and 2) the durable power of attorney for healthcare, or “healthcare proxy”, a document that assigns a surrogate to make medical decisions on behalf of a patient in the event of decisional incapacitation [2]. In general, discussions regarding ACP are shown to stimulate end-of-life conversations, reduce stress of surrogate decision-making, improve the quality of end-of-life care, and reduce life-sustaining therapies that are discordant with patient preferences [3-5]. Despite these potential benefits of ACP, recent evidence suggests that as few as 26% of American adults have completed an advance directive [6]. For those who did not complete an advance directive, lack of awareness was the most frequently reported barrier. Among young adults, the specific rate of ACP is unknown.

Young adults are a unique age group as they are still in the process of psychological development and identity transitions (i.e., individuation) [7]. Morbidity and death among peers are not common in this age group, partly due to the general wellness among this demographic [8]. When deaths do occur, sudden and unexpected trauma-related events are the leading cause of mortality among those ages 15–24 years [8]. Death from cancer in young adulthood follows as the leading cause of non-trauma-related death [9]. When a young adult is faced with a life-limiting or life-threatening condition, planning of end-of-life care preferences does occur, although in limited frequency [10,11]. The role of ACP for young adults is not yet fully articulated; nevertheless, the American Academy of Pediatrics, the Institute of Medicine, and the World Health Organization recommend involvement of adolescents and young adults in care decisions and ACP as early as they are developmentally and emotionally ready [12-14].

There is a significant gap in the literature regarding ACP and healthy young adults. To date, public education and advocacy efforts regarding ACP have largely targeted older adults or those with serious illnesses. Research regarding ACP among young adults has almost exclusively been limited to individuals with life-limiting illnesses (e.g., HIV, cystic fibrosis) [10,11,15-17]. Existing research among healthy young adults has focused on exploring preferences for hospice care—a specific end-of-life service—as opposed to the variety of options that comprehensive ACP entails [18,19]. Although research has described ACP preferences among healthy adolescents [20], we are interested in the young adult population that has full legal capacity. In order to understand healthy young adults’ perceptions of ACP, we conducted a focus group study of 18-30-year-old individuals recruited from the general public (i.e., not purposively recruited due to illness). Our research question was: “What are the knowledge, attitudes, and perceptions of generally healthy young adults regarding ACP?” This study serves as a first step in exploring the knowledge of this population regarding ACP, their readiness to learn and act, and their views and needs toward this proactive health behavior.

## Methods

Given the paucity of knowledge regarding healthy young adults and ACP, we chose a focus group design to gain a broad understanding of this population’s perspectives. In contrast to individual interviews, focus groups allow for dynamic interaction and collective learning between participants [21]. The institutional review board of the University of Pittsburgh approved our study protocol (#05-11091). All participants provided informed consent prior to study procedures.

### Participants

This study was performed at two universities in Pittsburgh, Pennsylvania. One university is a public university with a diverse urban campus that draws commuter students from outlying suburban communities, whereas the other is a private university. We recruited a convenience sample [22] through advertisements in campus newspapers and through advertisements on www.Facebook.com that were restricted to students from either of the two universities. Participants were eligible if they were current undergraduates or graduate students, and between 18–30 years old. Participants received $20 compensation and dinner during the session.

### Data collection

One author (DK) conducted six focus groups between February and April 2007. Group sessions lasted approximately 90 minutes each. We assigned participants to groups based on gender and age to promote diverse discussions within each session.

Participants completed a survey before the discussion assessing four domains: 1) demographics, 2) level of prior exposure, 3) baseline knowledge of advance directives, and 4) prior completion of ACP. Prior exposure was assessed through two questions: “Have you ever had a serious illness that required hospitalization, or continued medical monitoring, and placed you at risk for death and/or serious illness?” and “Has anyone in close contact to you (i.e., family, friends) experienced a serious illness that required hospitalization, or continued medical monitoring, and placed them at risk for death and/or serious illness?” Lastly, we asked participants to provide a free-text definition of an advance directive.

We developed a semi-structured discussion guide based on a literature review regarding ACP knowledge and preferences (Table 1). First, we elicited participants’ general attitudes towards healthcare decision-making, including barriers and facilitators. We asked participants to discuss their general perceptions of ACP, such as its purpose, benefits, and drawbacks. From there, we engaged participants in a discussion regarding their preferences for surrogate decision-making. For those interested in designating a surrogate, we inquired about the qualities and behaviors of an ideal surrogate. Lastly, we asked participants what types of information about ACP that they believed they and their peers would benefit from learning, and how they believe this information would be best disseminated.

**Table 1:** **Semi-structured interview guide: domains of interest and sample questions**

**Domain of interest**	**Sample question**
**Ice-breaker exercise**	“What does the term ‘autonomy’ mean to you?”
**Attitudes regarding medical decision-making**	“How comfortable do you feel making serious decisions about your own health care?”
	“What would make you more comfortable?”
	“Why do you believe that advance healthcare planning exists?”
	“When, if ever, do you believe that it is important to make your healthcare wishes known?”
**Preferences for surrogate decision-making**	“Would you prefer to make your own healthcare decisions, or would you prefer that someone else make them on your behalf?”
	“How do you believe that a surrogate comes to a decision on your behalf?”
	“How would you prefer that a surrogate would come to a decision on your behalf?”
**Preferences for ACP promotion**	“How would you prefer to learn more about advance care planning?”
	“What kinds of things do you think would be helpful for you and your peers to know about advance care planning?”

We approached quality control in a number of ways. First, a multidisciplinary team developed the interview guide. Second, our senior author, a health services researcher with expertise in mixed methods periodically reviewed transcripts during the course of the study to ensure the moderator’s interviewing quality (e.g., use of probes, cadence, tone). Third, the moderator regularly met with the senior author to debrief. Lastly, our study design, analysis, and reporting adhere to the RATS guidelines for qualitative research, which focus on research relevance, appropriateness of qualitative methods to answer the research question, transparency of procedures, and soundness of interpretive approach [23].

### Qualitative analysis

We used template analysis, a qualitative approach that combines content analysis and grounded theory [24]. Template analysis flexibly integrates a priori assumptions and hypotheses for a hybrid inductive/deductive analytic process. Data analysis was performed iteratively, with the initial codebook prepared from an extensive literature review. Within 48 hours after every focus group, audio recordings were transcribed verbatim, and we verified transcripts against the original recording to ensure transcription accuracy. Transcripts were compared with the moderator’s field notes to further ensure completeness. Within five days of each focus group, two investigators (DK and TKO) coded each transcript independently. Using the constant comparative technique [25], text units were compared with previously coded data to ensure stability and relevance of themes. Coding meetings were held until coders reached full consensus on definitions and application of codes. We determined that thematic saturation, the point at which no new themes, patterns, or relationships emerge from the data [26,27], occurred after the fifth group; we conducted one additional group to ensure saturation. Qualitative analysis was performed through a process of matrix and compound query retrieval using NVivo (QSR International, Doncaster, Australia).

### Demographic survey analysis

We analyzed survey data with descriptive statistics using Stata/IC (StataCorp, College Station, TX). We conducted a content analysis of the free-text definitions of “advance directive” provided by participants in their surveys. Two coders independently categorized these responses using a pre-specified taxonomy developed by the study team.

## Results

We conducted six focus group sessions involving a total of 56 young adults (14 men and 42 women, 6–13 per group). Participants were between 18 and 30 years old (median age, 21). Forty-three percent (n = 24) of participants were non-white (Table 2). One-tenth (11%, n = 6) of our participants expressed “primary exposure” (i.e., personal experience with critical illness), and 64% (n = 36) met our definition of having “secondary exposure” (i.e., critical illness and/or death of a close contact).

**Table 2:** **Participant demographics, and prior experiences with and preferences for advance care planning**

**Characteristic**	***n*** **(%)**
	**N = 56**
Mean age (range)	21 (18–30)
Female gender	42 (75)
Religious affiliation	
Christian	36 (64)
Atheist	4 (7)
Jewish	3 (5)
Other	13 (23)
Race	
White	40 (71)
African-American	8 (14)
Asian and Pacific Islander	8 (14)
Mean years of education	15 (i.e., undergraduate junior)
Planning to enter a helping profession^a^	26 (46)
Exposure	42 (75)
Primary^b^	6 (11)
Secondary^c^	36 (64)
Cancer-related	18 (32)
Unexposed	14 (25)
Prior experience with ACP^d^	20 (36)
Discussion with parent	15 (27)
Discussion with partner	5 (9)
Discussion with friend	2 (4)
Discussion with physician	1 (2)
Discussion with other relative	1 (2)
Created an AD	1 (2)
Designated a health care proxy	1 (2)
No prior experience with ACP	36 (64)
Participants interested in creating an AD^e^	41 (73)
Participants interested in designating a surrogate^d^	37 (66)
Mother	10
Either parent	9
Sibling	8
Father	5
Non-spouse partner	3
Other relative	1
Friend	1
Not interested in surrogate designation	19 (34)

Legend: ACP = advance care planning, AD = advance directive.

^a^The following disciplines were defined as helping professions: medicine, pharmacy, mental health, social work, rehabilitative sciences, and public health.

^b^Defined as an affirmative response to: “*Have you ever had a serious illness that required hospitalization, or continued medical monitoring, and placed you at risk for death and/or serious illness?”*.

^c^Defined as an affirmative response to: “*Has anyone in close contact to you (family, friends, etc.) experienced a serious illness that required hospitalization, or continued medical monitoring, and placed them at risk for death and/or serious illness?”*.

^d^Totals may exceed 100% due to subjects’ ability to select multiple options.

^e^Subjects were asked if they were interested in creating an advance directive and/or a health care proxy document within six months of the focus group interview.

### Baseline knowledge of advance directives

Most often, participants expressed that advance directives are a repository of treatment preferences (n = 31, 62%), activated when a person is unable to make their own decisions (n = 24, 48%). The most common misconception regarding advance directives was that they are a financial Last Will and Testament (n = 12, 21%).

### Qualitative themes regarding advance care planning

Given the focus group design of our study, the themes we present hereafter generally reflect the experience of multiple individuals; nevertheless, we also include perspectives shared by fewer individuals to document particularly salient viewpoints.

### Attitudes regarding medical decision-making

#### The value of autonomous decision-making

Participants often described a perceived need or expectation of being self-reliant. Although some participants intertwined self-determination with the fact that they had relatively recently been granted adult legal standing, most participants felt that all capable adults should undertake some form of ACP:

I definitely feel very comfortable [creating an advance directive] because I think I am a real person. I am considered legal in the US to drink. I can smoke, so why can’t I make an advance directive?

I feel most comfortable making this decision for myself, rather than having … my parents [decide] for me because, I mean, no one knows you better than you know yourself. So despite how uncertain you are or unwilling you might be to make these hard decisions, it’s far better than having someone else make these decisions for you that you don’t agree with.

For others, the need to be entirely self-determined was less important, and they were comfortable affording latitude to surrogates:

… I know kind of an idea of what I want and my fiancé has a general idea of what I’d want. … I’m kind of leaning toward that he would prolong [my life] longer than I would wish. But, that doesn’t really bother me in any way. If they’re his wishes, then they’re my wishes, too.

#### Age as a moderating factor

Participants in all groups freely discussed their preferences for ACP. Nevertheless, some participants seemed uncomfortable entertaining the possibility of incapacitation in the near future:

Moderator: “Let’s say that I were to give you the opportunity right now to create an advance directive. Would you [complete one]?”

Participant: “Hell no! At this point in my life, I don’t want to be thinking about death.”

Although age was mentioned as a motivator for some participants, others saw youth as a protective factor against the need to perform ACP.

I definitely could not [plan] now. I just can’t plan for my own death now. … I’m too young. I’m just too healthy now. I guess what it would take for me to do it would be a brush with my own death and not even one of a family member. … I guess I would just really have to feel that my death were close.

#### The role of social independence

Participants in each group discussed the role of interpersonal relationships on their perceived need to conduct ACP. We frequently heard participants speak of triggers for ACP. Most often, these triggers related to the process of individuating from their family of origin, and living more independently than when they were minors. Conversely, some participants felt that without a romantic partner or children of their own, their newfound social independence obviated their need for ACP:

… I probably won’t [complete an advance directive] because I don’t really think that I have a need to, as horrible as that sounds. … Nobody really thinks that their life depends on me, so I don’t have that much need for it.

#### The role of prior exposure

Participants spoke of the heightened need for ACP in light of serious illness. Participants who were the most vocal were those who had recent experiences with illness or death:

I would take you up on [creating an advance directive]. … [M]ainly because I lost my sister last year, and my mother’s had a really hard time with it.

### Preferences for surrogate decision-making

#### Family members as surrogates

Participants in all groups discussed their preferences for familial involvement in decision-making. Primarily, such discussions focused on participants’ parents; however, participants who were in long-term relationships sometimes mentioned their preferences for partner authority. Several participants desired a combination of parental and partner surrogates, expressing their hopes that concordance could be reached:

I think that at this point in my life, I don’t feel like I have a responsibility to write that down because if [incapacitation] would happen to me, it wouldn’t be a particular burden on anyone but my parents and my fiancé, and maybe they could work it out together?

Other participants were uncomfortable with parental surrogates, distrusting a parent’s ability to remain objective:

I don’t particularly want my parents making my decision for me because I think that their judgment would be clouded because, you know, I’m their little girl. I’m their daughter; they adore me. I need someone more objective than that because, you know, parents try to beat the odds.

### Preferences for ACP promotion

#### Appeals for more information

Participants in all groups were interested in more information on ACP options, even those who were not necessarily interested in completing an advance directive at the time of the group.

… [I]f I was more educated on specific healthcare policies or whatever, I think that I’d be more comfortable making these decisions. At this point in my life, I feel like I know what’s important to me and I know enough about myself to make that type of decision.

## Discussion

To our knowledge, this study is the first in-depth analysis of the knowledge and preferences of healthy young adults regarding ACP. Though other studies have investigated healthy young adults’ perspectives on hospice care [19], our broader examination of ACP provides a richer understanding of how ACP may be operationalized for this population. Some might argue that introducing ACP in healthy young adults is inappropriate given decisional inconsistencies, such as the inability to envision oneself in a future health state [28,29]. Although ACP for healthy young adults may seem premature, the fact that all of our participants wanted more information about ACP speaks to the appeal of educational intervention. Acquainting young adults with ACP topics may aid them in making decisions for their elders, in addition to their own eventual ACP process, even if it does not occur in the immediate future.

Our findings are consistent with guiding theory in ACP research. Multiple groups have published on the applicability of the stages of change models to behavior change in ACP, such as the Transtheoretical Model [30-33]. In the Transtheoretical Model, individuals progress through five stages (i.e., precontemplation, contemplation, preparation, action, and maintenance) [34]. Our finding that most young adults know very little about ACP, but are willing to learn more places them in either the precontemplation and contemplation stage, respectively. Those in precontemplation have not been introduced to ACP or do not want to engage in ACP activities. Likewise, the participants in contemplation were willing to gather information about the purpose of the behavior and think about prioritizing it in aspects of their own lives. Our findings are in line with work by Fried and colleagues, who demonstrated that roughly half of individuals aged sixty-five and older are in either the precontemplation or contemplation stages regarding ACP [31]. In the present study, we observed that the proportion of young adults in the precontemplation and contemplation stages is even greater than that in older adults. This finding is reasonable given the relatively higher frequency with which ACP is discussed and applied in the latter group.

To date, much of the literature regarding ACP has focused on older adults. Adding data on young adults is needed. First, a lack of young adult ACP educational interventions deprives educational opportunities for those who will likely act as surrogate decision makers for their elders. Second, ACP education may help young adults conceptualize their own end-of-life circumstances, helping them through the process of ACP along the life-course.

Young adults in our study exhibited significant misunderstandings regarding ACP. Paired with a unanimous desire to learn more, this may indicate a role for public health to educate young adults regarding ACP. Even if young adults choose not to perform ACP immediately, exposure to information may facilitate ACP when deemed appropriate. Further, this act of priming may promote relevant discussions with their contacts, thereby potentially increasing the likelihood of preferred outcomes for both themselves and for those whom they may serve as surrogates. We identified a subset of participants who, after learning more, were quite eager to engage in ACP themselves. These findings suggest the potential benefit of an educational intervention to prepare this cohort as surrogate decision makers, while simultaneously providing a prelude to their own ACP process.

Several limitations merit comment. First, our sample was a convenience sample derived from one city; therefore, the perspectives captured herein are not generalizable to all young adults. Further, our sample may not represent the overall young adult population regarding education and socioeconomic status (all participants had completed at least some college), both of which may influence engagement in healthcare decision-making. As such, our findings may be an overstatement of ACP knowledge in the overall young adult population. It is reasonable to hypothesize that individuals with less education and less concerned with healthcare may have reduced comprehension of ACP definitions and goals. Lastly, these data are from 2007; nevertheless, they address an important gap in the literature, as this remains the first study to broadly explore young adults’ knowledge, attitudes, and preferences regarding ACP.

Future research should seek to identify young adult-centered models of shared decision-making, which reflect one’s developmental stage and readiness to undertake ACP for themselves or—more likely—as surrogates for older generations. Such models of ACP must be flexible to allow for goal alteration over time. Given our findings, young adults may prefer to become informed and work through states of readiness culminating (perhaps years later) with their own advance directive or designation of a healthcare proxy.

Appeals have been made for public health to promote population-based healthcare engagement through ACP [35,36]. A recent commentary in the *American Journal of Public Health* argues that empowering individuals to engage in ACP is clearly within the purview and mission of public health [35]. Nevertheless, it remains that providing knowledge and communication regarding ACP is a necessary first step to promoting this health behavior. Future research is needed to understand the mechanisms by which public health can engender meaningful healthcare decision-making among all facets of the population, regardless of age or health status.

## Conclusions

This study offers an initial examination of knowledge, attitudes, and preferences of healthy young adults regarding ACP. Our findings suggest that 1) relatively few young adults fully understand ACP concepts, and 2) all young adults in our sample desired to learn more about ACP. The confluence of these two factors presents a gap that public health practice is well-suited to fill, beginning with educational interventions. Our work provides a roadmap for understanding how to operationalize recent calls for the public health community to promote population-based healthcare decision-making [35,36].

## Abbreviation

ACP: Advance care planning
